# Round the Hospitals

**Published:** 1921-04-23

**Authors:** 


### ROUND THE HOSPITALS.

The regulations of the General Nursing Council for Ireland governing the admission of "existing" nurses to the General Register and its Supplementary Parts are now published, and nurses desirous of being admitted to the Irish Register should at once take advantage of them and apply for forms of application to the Registrar. Training in a general hospital or Poor-law Infirmary, either for three years, or for one year followed by two years' *bona-fide* practice, is essential for registration, save in the case of the "qualified" nurse, who however must show she has been employed with the approval of the Local Government Board for Ireland in this capacity for at least three years. To what extent the latter class of nurses will apply for registration only time can show. The term covers "any person who after examination has obtained a certificate of proficiency in nursing from any (1) public general hospital; or (2) workhouse infirmary and fever hospital; or (3) nursing institution." To deny them registration after officially labelling them as "qualified" was perhaps impossible, but we fear that in many instances their opportunities for acquiring a sound knowledge of nursing have been very scanty. It is hard to believe that there are many nurses whose period of hospital or infirmary training is measured by one year, for it has now for a very long time been exceedingly difficult to procure one year's training only.

As the registration rules framed by the English, Scottish and Irish Councils begin to appear the question which dominates the hour is the extent to which they are in harmony one with another. Will there be practically three standards for the United Kingdom? Or will nurses trained and registered in one part of the country be at liberty to obtain registration automatically in whatever part of the kingdom they may desire to practise? In the *Irish Times* of the 1st inst. we read that "nurses admitted to the Irish Register will be automatically entitled to admission to either the English or Scottish Register if they should go to Great Britain to practise, and the Irish Council will accept nurses already registered in England or Scotland." No doubt the wish is father to the statement; but has the matter been fairly envisaged by the three Boards? It would be a miracle if all three Boards, acting entirely independently, had evolved a general standard of efficiency equal in all particulars. And let it be granted that one or other set of the rules for registration were to prove more lax than that of the others, is it not perfectly clear that automatic mutual registration would involve somewhat of an injustice?

The regulations in respect of the new Royal Air Force Nursing Service, established by Royal Warrant in January last, are now issued. The Service consists of matron-in-chief, matrons, senior sisters, sisters, and staff nurses. Candidates for the post of staff nurses must be of British parentage, fully trained, and between the ages of twenty-five and thirty-five. Salaries are as follow: Staff nurses, £60, rising by yearly increment of £2 10s. to £65; sisters, £75, rising yearly by £5 to £85; senior sisters, £85, rising yearly by £10 to £95; matrons, £115, rising yearly by £10 to £185. Charge pay not exceeding £45 is granted to matrons according to the magnitude of the charge. The allowances consist of 24s. 6d. per head in lieu of board and washing, in addition to furnished quarters, fuel, light, and attendance, or an equivalent allowance in money. Uniform allowance, £20 on appointment, £5 for second year, and £10 every subsequent year. Retirement is optional either after twenty years' service or at the age of fifty, and is compulsory at fifty-five. Retired pay, based on service and rank, is issued up to the maximum rate: Matrons, £170; sisters, £75; staff nurses, £55. Previous time spent in the temporary Air Nursing Service and the Navy or Army Nursing Service counts towards retired pay. All particulars and forms of application to be obtained from the Matron-in-Chief, Air Ministry, Kingsway, W.C. 2. We understand that nurses with the certificates of the C.M.B. and the C.S.M.M.G. will have priority in selection. The nursing service of the wards available in some of the hospitals for the wives of members of the R.A.F. necessitates the former, and in general massage is likely to be a frequent requirement.

A proposal has been put forward at Manchester in connection with the Poor-law Officers' Association to found a Poor-law Nurses' Guild. The number of nurses who have enrolled in the Poor-law Officers' Association has never satisfied the Executive Committee, and even the provision of special "nursing sections," designed to constitute a nursing association within the main Association, has not entirely met the needs of Poor-law nurses. The new Guild at Manchester will, in effect, form one of these Nursing Sections. Membership is confined entirely to members of Poor-law infirmary nursing staffs, who will elect their own officers and be entitled to representation on their own branch of the parent Association. It was expressly stipulated by Miss Burgess, matron of Crumpsall Infirmary, that there should be no question of the National Association becoming a trade union, and on that understanding the Manchester and District Poor-law Nurses' Guild came into being, with Miss Burgess for Chairman, Miss Ashton Secretary, and a strong Provisional Committee with power to accept as honorary members any medical men who are members of the Poor-law Officers' Association.

The nursing staff of the London Hospital are making an effort to throw off the shackles of the Unemployment Insurance Act. Sixty-five sisters, 145 private staff nurses, and 287 staff nurses and probationers of the London Hospital have spontaneously drawn up and signed a protest against the inclusion of nurses in the Act, and this protest has

**ROUND THE HOSPITALS—(*continued*).**

been sent to the Minister of Labour. It came entirely from the sisters and nurses themselves, and was in no way suggested by the matron. This desire for exclusion on the part of the London Hospital nurses appears to be typical of that felt by the large body of nurses throughout the country. It should be borne in mind, however, that the decision of the Labour Minister, announced in these columns on March 26, applies only to probationers and nurses in a voluntary hospital. The position of sisters under the Act is still under consideration, and a decision has not yet been given, whilst the position of private nurses also remains undecided.

We are asked to state that the Conference on Nurse-Training of hospital matrons and nursing experts which is to be held under the auspices of the General Nursing Council at the rooms of the Royal Society of Medicine, 1 Wimpole Street, W. 1, on April 28, will be private and not open to the Press. As soon as possible after the meeting, an official communication will be issued to the Press for publication. The conferences are timed to begin at 11 A.M. and 2.30 P.M., and the doors will be open at 10.30 A.M. and at 2 P.M.

The nursing profession has lost a representative of its highest traditions, and the Royal Hospital, Richmond, a devoted and able matron in Miss A. L. Yelverton Dawson, who died on April 8 at St. Thomas's Hospital. Miss Dawson trained at the Nightingale Training-school, St. Thomas's Hospital, from 1898 to 1901, and was then appointed sister-in-charge of "Beatrice" ward; five years after she was made sister housekeeper and in charge of the scrubbers, and was known as "Sister Louise." She was appointed matron of the Royal Hospital, Richmond, in 1908, and held that post till her death. During the war some eight hundred wounded soldiers were treated at Richmond; the resident medical staff was seriously depleted; and the difficulties incidental to the smaller hospitals in the circumstances of the time had to be met. Miss Dawson was always ready to deal with every emergency, and she herself undertook much of the casualty work under the supervision of the visiting staff. Her splendid work then and at all times will long be remembered by all who knew her. At the funeral service held at St. Thomas's Hospital on April 12, amongst many colleagues and old and new friends, were twelve scrubbers who had worked under her as Sister Louise.

Nursing problems in the United States present features similar in all respects to those under which we labour in this country, and never has this been brought more forcibly to view than in the Nursing Survey of Cleveland, Ohio, compiled by Miss Josephine Goldmark, as part of the comprehensive Hospital and Health Survey of that city, just issued. Lack of good candidates, defective affiliation, deficiencies in the training-schools both as regards instructors and equipment, financial difficulties—these are some of the causes which retard nursing development in Cleveland. Miss Goldmark recommends the establishment of a "university" training-school, giving a definite period of college training, an equal length of time for hospital training, and a final academic period for additional courses in nursing specialities. She also urges the establishment of a compulsory minimum educational requirement, and the provision of ward helpers with a view to shortening the three years' hospital course. At Cleveland all the various services depending for their existence on nursing are co-related under a Central Nursing Committee, and the result is to diminish friction and overlapping, besides maintaining a high standard of nursing care in every direction. In this respect we have much to learn.

